# Plasma metabolomic study in Chinese patients with wet age-related macular degeneration

**DOI:** 10.1186/s12886-017-0555-7

**Published:** 2017-09-06

**Authors:** Dan Luo, Tingting Deng, Wei Yuan, Hui Deng, Ming Jin

**Affiliations:** 1 Department of Ophthalmology, China-Japan Friendship Hospital, Yinghua Donglu, Chaoyang District, Beijing, 100029 China; 2 Beijing Changping Hospital of Chinese Medicine, South section of East Ring Road, Changping District, Beijing, 102200 China; 3 Institute of Clinical Medical Sciences, China-Japan Friendship Hospital, Yinghua Donglu, Chaoyang District, Beijing, 100029 China

**Keywords:** Age-related macular degeneration, Metabolomic study, Amino acids, Metabolites

## Abstract

**Background:**

Age-related macular degeneration (AMD) is a leading disease associated with blindness. It has a high incidence and complex pathogenesis. We aimed to study the metabolomic characteristics in Chinese patients with wet AMD by analyzing the morning plasma of 20 healthy controls and 20 wet AMD patients for metabolic differences.

**Methods:**

We used ultra-high-pressure liquid chromatography and quadrupole-time-of-flight mass spectrometry for this analysis. The relationship of these differences with AMD pathophysiology was also assessed. Remaining data were normalized using Pareto scaling, and then valid data were handled using multivariate data analysis with MetaboAnalysis software, including unsupervised principal component analysis and supervised partial least squares-discriminate analysis. The purpose of the present work was to identify significant metabolites for the analyses. Hierarchical clustering was conducted to identify metabolites that differed between the two groups. Significant metabolites were then identified using the established database, and features were mapped on the Kyoto Encyclopedia of Genes and Genomes.

**Results:**

A total of 5443 ion peaks were detected, all of them attributable to the same 10 metabolites. These included some amino acids, isomaltose, hydrocortisone, and biliverdin. The heights of these peaks differed significantly between the two groups. The biosynthesis of amino acids pathways also differed profoundly between patients with wet AMD and controls.

**Conclusions:**

These findings suggested that metabolic profiles and and pathways differed between wet AMD and controls and may provide promising new targets for AMD-directed therapeutics and diagnostics.

## Background

Age-related macular degeneration (AMD) is at present one of the most important diseases leading to vision loss and blindness in the elderly. The number of people with AMD is projected to increase from 196 million in 2020 to 288 million in 2040 [1]. According to clinical presentation, late-stage AMD has two types: dry (atrophic) AMD and wet (neovascular or exudative) AMD. In particular, wet AMD is characterized by subretinal neovascular membrane or choroidal neovascularization, resulting in subretinal hemorrhage, yellowish-white effusion, detachment of retinal pigment epithelium, and the formation of fibrous vascular scar. Wet AMD comprises only 10–20% of all AMD, but 80–90% of severe visual impairment is caused by wet AMD [2, 3]. In China, survey data shows that the prevalence of AMD increases annually and is currently escalating at a rate of 15.5%, of which 11.9% is wet AMD [4].

As reported previously, many treatment methods, such as intravitreal anti-vascular endothelial growth factor injection and photodynamic therapy [5, 6], have been adopted for AMD; however, effective therapeutic strategies for curing AMD have yet to be developed. Therefore, aside from inflammation, oxidative stress, and genetic predisposition, the complex pathophysiology of AMD must be explored further. Other factors, such as age, diet, and smoking status, may also be risk factors for AMD [7–9]. Metabolomics can be used to comprehensively analyze the complex metabolic substances of biological systems during particular stages and so indicate the metabolic changes induced by internal and external causes through qualitative and quantitative detection of these components [10–12]. In recent studies, metabolic disturbance has also been found to be strongly associated with AMD, and certain metabolic pathways, such as some amino acid, organic acid, and urea metabolism, might be involved in AMD pathogenesis [13, 14]. In the metabolomics of retinal degeneration, a number of classes of metabolites, such as vitamin A analogues, fatty acid amides, long-chain polyunsaturated fatty acids, acyl carnitines, and several phospholipid species, were consistently dysregulated during degeneration [15]. Nutritional and lifestyle habits may also affect the metabolic state in AMD [9, 14]. An animal study of nontargeted metabolomics showed that microbial cometabolites could confer protection against AMD [9]. A study from Japan on the association between nutrient intake and wet AMD confirmed low intake of n-3 fatty acid, α-tocopherol, zinc, vitamins D and C, and β-carotene to be associated with wet AMD [16]. In this way, in the current study, ultra-high-pressure liquid chromatography and quadrupole-time-of-flight mass spectrometry (UHPLC-Q-TOF MS) techniques were used to detect metabolic differences between Chinese patients with AMD and controls to assess the pathogenesis of and determine appropriate treatment for AMD.

## Methods

### Ethics statement

This case–control study was approved by the Ethics Committee of the China-Japan Friendship Hospital and was conducted in accordance with the provisions of the Declaration of Helsinki. Written informed consent was obtained from all participants prior to enrollment in the study.

### Participants

Patients over 50 years old were enrolled from the Outpatient Department of Ophthalmology at the China-Japan Friendship Hospital and were diagnosed with wet AMD in one or both eyes [17]. Healthy volunteers without clinical presentation of AMD were recruited as controls. Wet AMD status was confirmed through direct ophthalmoscopy, optical coherence tomography, fundus fluorescein angiography, and indocyanine green angiography. All participants had normal results from routine tests for urine and blood, liver and kidney function, and blood glucose and blood lipids. Smoking habits and special dietary supplement history were recorded from all participants. Exclusion criteria included ocular fundus diseases other than AMD, such as uveitis, central serous chorioretinopathy, Stargardt disease, diabetic retinopathy, and arteriosclerotic retinopathy.

### Sample collection

After enrollment, approximately 4 ml blood was extracted from the cubital vein of each participant after more than 12 h of fasting. The blood was then immediately transferred to the collection tube. All tubes were centrifuged at 3000 rpm/min for 10 min at 4 °C to remove blood cells, and plasma was immediately frozen at −80 °C prior to metabolomic detection.

### Metabolomic analysis

Frozen plasma samples from 20 wet AMD cases and 20 controls were thawed and analyzed through UHPLC-Q-TOF MS combined with data-independent acquisition method to perform full spectrum analysis and concurrently obtain primary mass spectrometry and secondary mass spectrum data [18].

### Sample pretreatment

A total of 100 μl plasma sample was treated with 400 μl methanol/acetonitrile (1:1, *v*/v) under vortex mixing for 30s and then placed at −20 °C for 10 min. After, plasma samples were centrifuged at 14,000 g/min for 15 min at 4 °C, and 380 μl supernatant was dried in a vacuum and kept in −80 °C. The quality control (QC) samples were prepared using the above methods. For mass spectrometry, the samples were treated with acetonitrile water solution (acetonitrile/water = 1:1 *v*/v) for detection.

### Gas chromatography–mass spectrometry (GC-MS) analysis

#### Chromatographic condition

All samples were separated through reversed-phase UHPLC with Agilent 1290 Infinity liquid chromatograph (Agilent, U.S.). Detection conditions were as follows: 25 °C column temperature; 300 μl/min flow rate; 2 μl sample size. The moving phase in cationic mode of HSS T3 chromatographic column (Waters, ACQUITY UPLC HSS T3 1.8 μm, 2.1 × 100 mm column) contained the following: A: 0.1% formic acid aqueous solution (Fluka, 06450); B: 0.1% formic acid acetonitrile. The moving phase in cationic mode contained the following: A: 0.5 mm ammonium fluoride solution; B: acetonitrile (Merck, 1,499,230–935). Gradient elution program was as follows: 1% B for 0–1.5 min; B linear change from 1 to 99% for 1.5–13 min; 99% B for 13–16.5 min; B linear change from 99 to 1% for 16.5–16.6 min; 1% B for 16.6–20 min. Samples were kept at 4 °C in the automatic sampler throughout the analysis. To avoid the influence of signal fluctuation of instrument detection, samples were analyzed in random order. Samples were placed in the sample queue in groups of 5, and one set of QC sample was used for monitoring and evaluating the stability of the system and the reliability of the experimental data.

#### Quadrupole–time-of-flight conditions

Detection was performed through electrospray ionization (ESI) cationic and anionic modes. Then samples were separated through UHPLC, and mass spectrometry analysis was performed using Triple 6600 TOF mass spectrometer (AB SCIEX). ESI source conditions were as follows: Ion Source Gas1 (Gas1): 40; Ion Source Gas2 (Gas2): 80; curtain gas (CUR): 30; source temperature: 650 °C; Ion Sapary Voltage Floating ±5000 V (cationic and anionic modes); TOF MS scan m/z range: 60–1000 Da; product ion scan m/z range: 25–1000 Da; TOF MS scan accumulation time: 0.20s/spectrum; product ion scan accumulation time: 0.05 s/spectrum. The secondary mass spectrometry was performed through information-dependent acquisition (IDA) [high-sensitivity mode; declustering potential: ± 60 V (cationic and anionic modes), collision energy: 35 ± 15 eV], and IDA was set as follows: exclude isotopes within 4 Da; candidate ions to monitor per cycle: 10.

### Data analysis

Descriptive analysis was used for the basic characteristics. Two-sample mean comparison for age was made using independent sample t-test. The two-tailed Fisher exact test was used for gender and comorbidities. Original metabolomic data were converted to mzML through ProteoWizard and then those data were handled with peak alignment, retention time adjustment, and extraction of the peak area for XCMS analysis. Structural identification was performed through mass matching (<25 ppm) and secondary spectrogram matching, which should be searched and compared using the lab database (Shanghai Applied Protein Technology Co., Ltd.).

Ion peak data handled with XCMS [19] were excluded when the missing value of two groups exceeded 50%. The remaining data were normalized through Pareto scaling, and then the valid data were handled using multivariate data analysis, such as unsupervised principal component analysis (PCA) and supervised partial least squares-discriminate analysis (PLS-DA), using MetaboAnalysis software. Concurrently, the valid data were handled with unidimensional statistical analysis, such as the t-test, variation ratio analysis, and volcano plot analysis through R software.

For metabolite annotation and pathway analysis, metabolites that showed differences between groups were selected and analyzed according to the multidimensional statistical analysis selection criteria and univariate statistical analysis selection criteria using the Kyoto Encyclopedia of Genes and Genomes (KEGG; http://www.genome.jp/kegg/) database.

## Results

### Clinical characteristics

This study was conducted on 20 patients with wet AMD (27 eyes, 11 men and 9 women, mean age 66.20 ± 11.51 years, mean duration 18.46 ± 17.55 months) and 20 controls without any clinical presentation of (11 men and 9 women, mean age 64.70 ± 11.60 years) AMD. No differences were noted between two groups in gender or age. All participants were Chinese and long-term residents of China.

The wet AMD group included two smokers (10.0%) and the control group had one (5.0%). Five (25.0%) of wet AMD patients took vitamin supplements, and three (15.0%) of controls did. These factors did not differ between groups. No significant difference was noted in the percentage of patients between two groups with respect to comorbidities, such as hypertension (9 wet AMD for 45.0%, 8 controls for 40.0%), coronary artery disease (2 wet AMD for 10.0%, 1 control for 5.0%), and hyperlipidemia (3 wet AMD for 15.0%, 2 controls for 10.0%).

### Inter-group PCA analysis

**Fig. 1:**
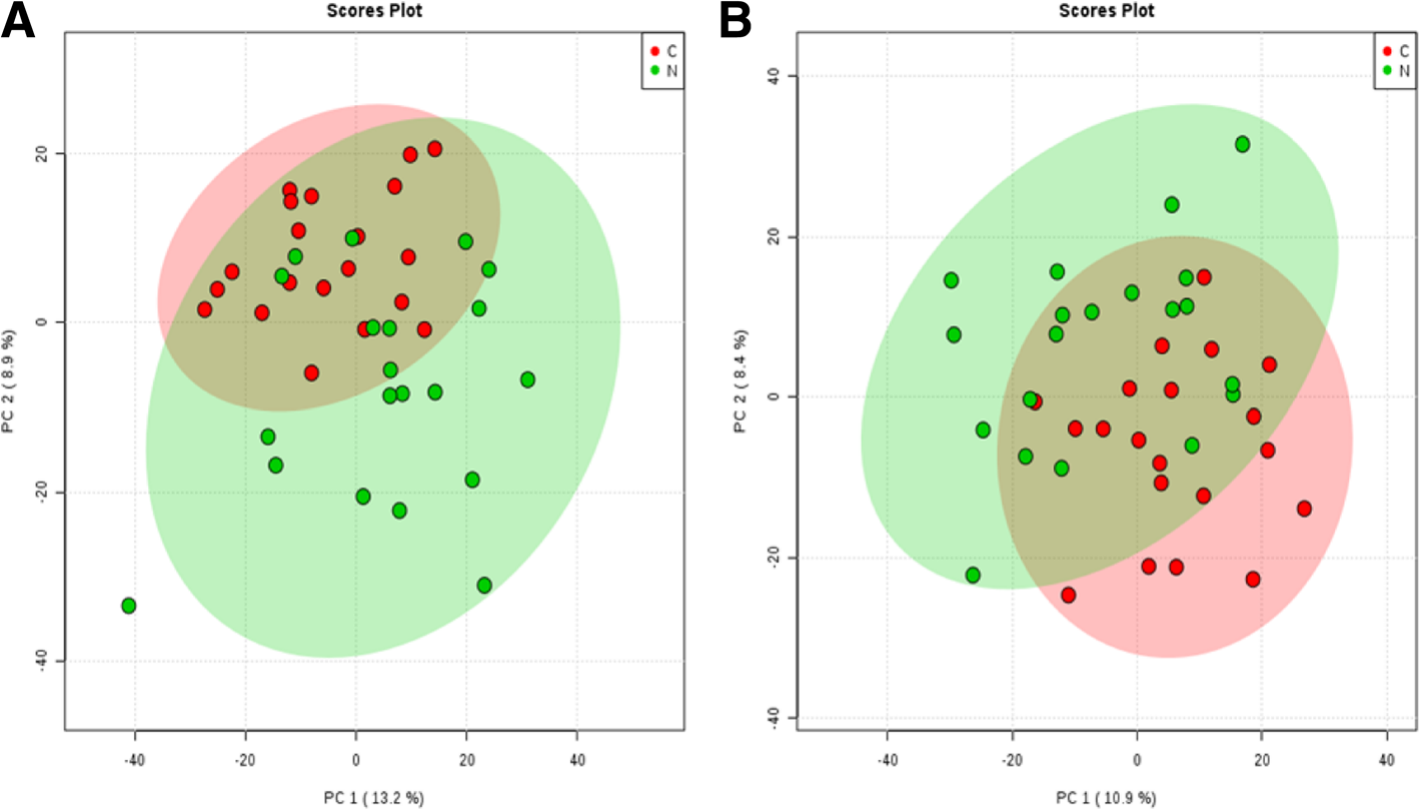
**a** PCA scores for wet AMD and control groups under the cationic mode. **b** PCA scores for wet AMD and control groups under the anionic mode. C: Wet AMD group; N: Control group

A total of 5443 ion peaks of metabolites were recorded using XCMS. After Pareto scaling, PCA, which can reflect the differences of metabolism between groups and variabilities in each group, was performed and indicated the real differences between groups. As shown in Fig. 1, on a PC1 and PC2 dimension chart, a trend of separation was visible between wet AMD and control groups, indicating that the spectrum of plasma metabolism in the two groups changed.

### Inter-group PLS-DA analysis

**Fig. 2:**
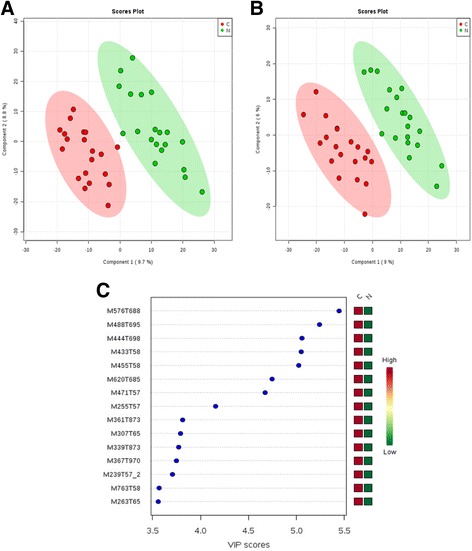
**a** PLS-DA with 2D score plot for wet AMD and control groups under the cationic mode. **b** PLS-DA with 2D score plot for wet AMD and control groups under the anionic mode. **c** Under the cationic mode, the top 15 different metabolites were screened through PLS-DA. C: Wet AMD group; N: Control group

We used supervised multidimensional statistical methods, specifically PLS-DA to analyze samples from the two groups and so obtain information about metabolites that differed significantly between the two groups. According to the model evaluation parameters, particularly under the cationic mode, R^2^ = 0.96 and Q^2^ = 0.65 > 0.5 indicated that the PLS-DA mode was stable and reliable and showed distinct metabolomic plasma profiles for the wet AMD and control groups (Fig. 2a); however, the Q^2^ = 0.45 is <0.5 under the anionic mode; thus, the stability and reliability of the PLS-DA mode was poor (Fig. 2b).

Under the cationic mode, a total of 5443 ion peaks were tested. Based on the above established PLS-DA mode, we examined different biologically significant metabolites. The variable importance for the projection (VIP) can measure impact strength and interpretation capability of each metabolite pattern to distinguish samples in each group. Taking VIP score > 1 as the selection criterion, 864 metabolites were preliminarily screened between groups. According to the data of HSS T3 cationic mode, PLS-DA mode screened the top 15 different metabolites (Fig. 2c).

### Inter-group volcano plot analysis

**Fig. 3:**
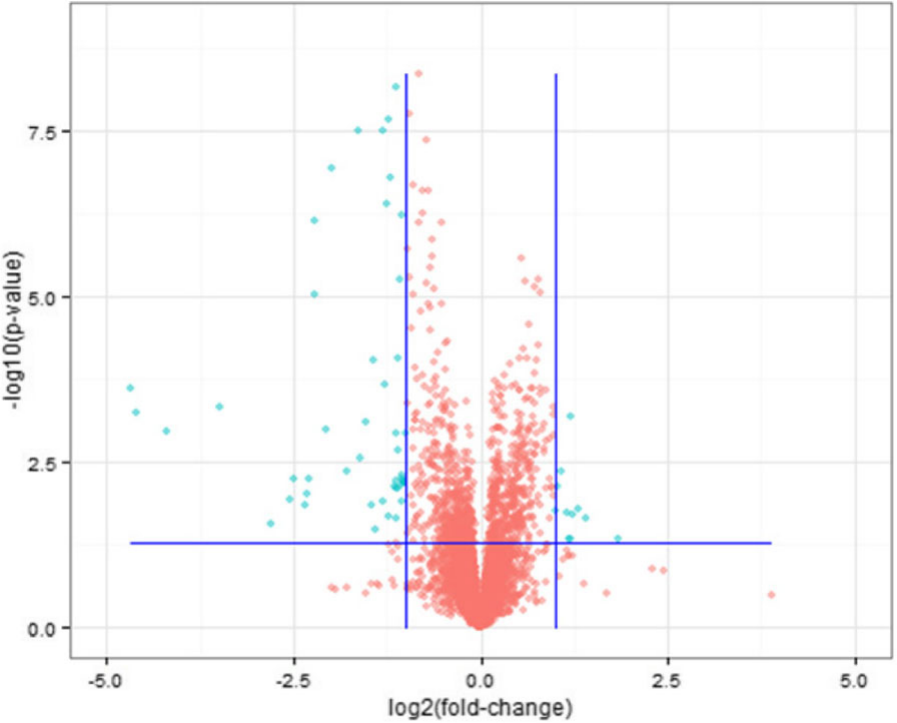
Volcano plot under the cationic mode, and blue points represent significant different metabolites (FC > 2.0 and *P* value <0.05)

Univariate analysis can visually indicate the significance of metabolite changes between the two samples. Here, Volcano Plot analysis synthesized Fold Change (FC) analysis and t-test, which can help screen potential metabolites. As shown in Fig. 3, under the cationic mode, taking FC > 2.0 and *P* value <0.05 as the selection criteria, the blue points show metabolites that differed between the two groups.

### Metabolites with significant differences between groups

We identified metabolites that differed significantly between the two groups using VIP > 1 and *P* value <0.05 or 0.05 < *P* value <0.1 as criteria. A total of 10 metabolites met these conditions (Table 1), particularly the amino acids, and this might indicate that the metabolic pathways of these amino acids are involved in AMD pathogenesis. Significant features were annotated in KEGG database using positive ion adducts (M + H or M + H-H_2_O).

**Table 1:** Metabolites with significant differences between groups

Adduct	Description	Fold change	*P*-value	VIP	m/z	rt s
(M + H)+	N-Acetyl-L-alanine	1.44	0.031	1.62	132.0651	62.14
(M + H)+	N1-Methyl-2-pyridone-5-carboxamide	0.67	0.033	1.73	153.0652	207.81
(M + H)+	L-Tyrosine	1.14	0.031	1.09	182.0812	66.74
(M + H)+	L-Phenylalanine	1.25	0.014	1.58	166.0857	241.44
(M + H)+	L-Palmitoylcarnitine	0.79	0.025	1.39	400.3424	619.41
(M + H)+	L-Methionine	1.12	0.038	1.08	150.0578	66.42
(M + H)+	L-Arginine	1.22	0.094	1.09	175.1186	49.01
(M + H-H_2_O)+	Isomaltose	1.22	0.010	1.34	325.1125	50.74
(M + H)+	Hydrocortisone	0.76	0.060	1.01	363.2165	426.53
(M + H)+	Biliverdin	0.71	0.011	1.43	583.2533	488.13

We used the amount of expression of qualitatively significantly different metabolites to evaluate the rationality of the candidate metabolites and to more fully and intuitively display the relationships among samples and differences in expression patterns via hierarchical clustering of each sample. This helped us accurately screen marked metabolites and study the changes in related metabolic processes.

**Fig. 4:**
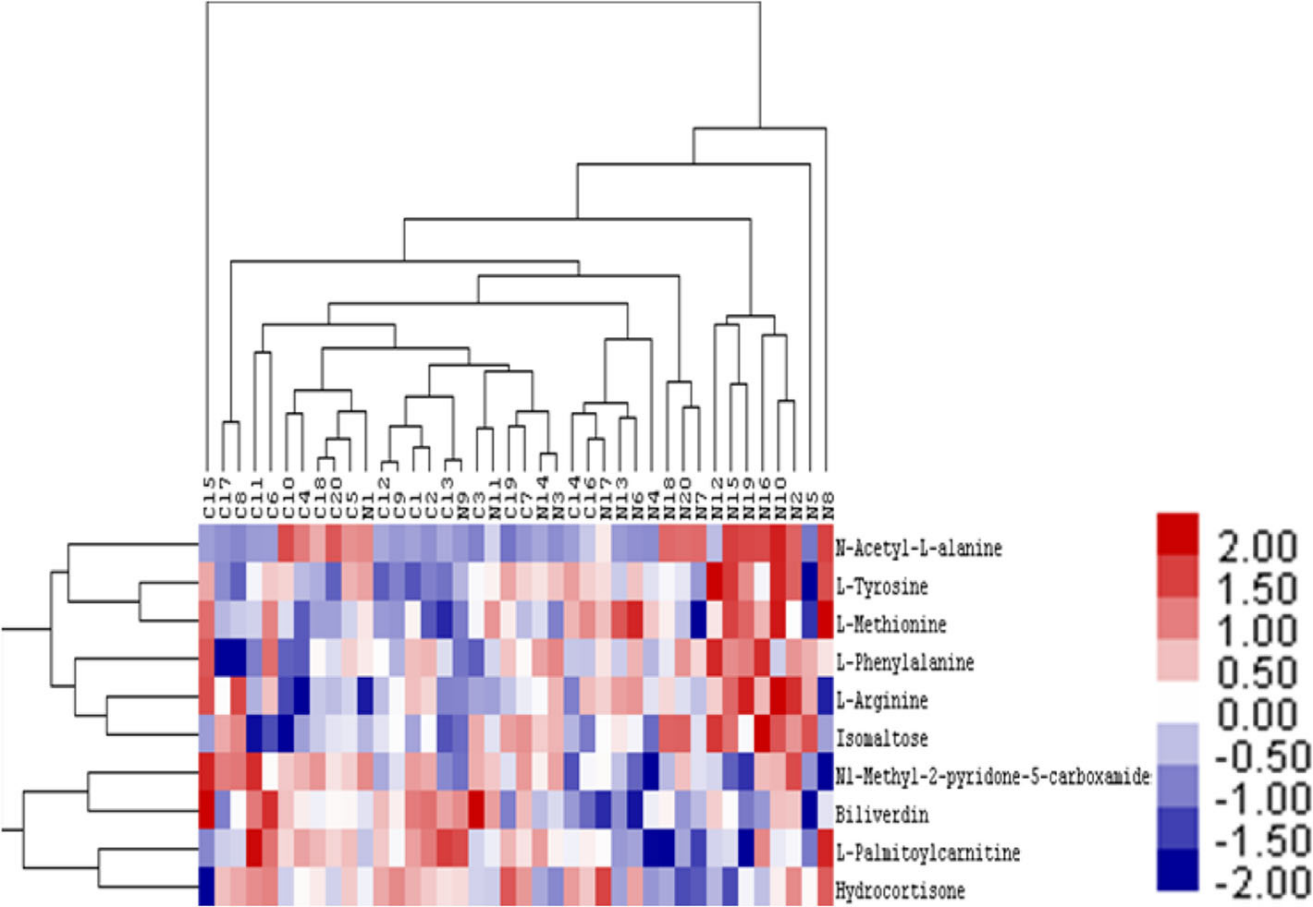
Hierarchical clustering of significantly different metabolites under the cationic mode. C: Wet AMD group; N: Control group

In general, when the screening of the metabolites was reasonable and accurate, the samples from one group appeared in one cluster. Metabolites gathered in the same cluster have similar patterns of expression, which may indicate that they are involved in adjacent or close steps in the overall process of metabolism. The tree structure on the left side of Fig. 4 represents the clustering relationships of each metabolite, and the tree structure at the top represents the clustering relationships of each sample. Hierarchical clustering results also showed that significantly different metabolites existed between two groups, although some metabolites also showed similarities.

### KEGG metabolic pathway analysis of different metabolites

A nontargeted metabolomic method was used in this study. Metabolites were screened and placed into KEGG database to determine chemical and metabolic pathways that could be involved in wet AMD. Because data processing included more than one metabolic pathway in the KEGG database, 68 possible pathways were found to be involved, covering 23 specific metabolites. Based on these KEGG human metabolic pathways, we searched for matching metabolic pathways within different metabolites. A total of 14 metabolic pathways were included, with ≥5 different metabolites (Table 2). As shown in Table 2, the biosynthesis of amino acid pathways included seven different metabolites, L-arginine, L-methionine, L-tryptophan, L-phenylalanine, L-tyrosine, L-proline, and N-acetyl-L-alanine. In addition to metabolic pathways involving amino acids, matches for sugar and steroid metabolic pathways indicated that these substances could also be involved in the pathogenesis of AMD.

**Table 2:** Affected metabolic pathways including more than ≥3 different metabolites (KEGG database matched results, C00062: L-arginine; C00073: L-methionine, C00078: L-tryptophan, C00079: L-phenylalanine, C00082: L-tyrosine, C00148: L-proline, C00624: N-Acetyl-L-alanine; C00408: L-pipecolic acid; C01487: D-allose)

ID	Metabolic pathway name	Cpd
map01230	Biosynthesis of amino acids (7)	C00062,C00073,C00078,C00079,C00082,C00148,C00624
map00970	Aminoacyl-tRNA biosynthesis (6)	C00062,C00073,C00078,C00079,C00082,C00148
map01130	Biosynthesis of antibiotics (6)	C00062,C00078,C00079,C00082,C00148,C00624
map05230	Central carbon metabolism in cancer (6)	C00062,C00073,C00078,C00079,C00082,C00148
map04974	Protein digestion and absorption (6)	C00062,C00073,C00078,C00079,C00082,C00148
map01060	Biosynthesis of plant secondary metabolites (6)	C00062,C00073,C00078,C00079,C00082,C00408
map01210	2-Oxocarboxylic acid metabolism (5)	C00073,C00078,C00079,C00082,C00624
map00966	Glucosinolate biosynthesis (4)	C00073,C00078,C00079,C00082
map04978	Mineral absorption (4)	C00073,C00078,C00079,C00148
map02010	ABC transporters (4)	C00062,C00079,C00148,C01487
map01063	Biosynthesis of alkaloids derived from shikimate pathway (3)	C00078,C00079,C00082
map01070	Biosynthesis of plant hormones (3)	C00073,C00078,C00079
map01064	Biosynthesis of alkaloids derived from ornithine, lysine and nicotinic acid (3)	C00062,C00079,C00408
map00400	Phenylalanine, tyrosine and tryptophan biosynthesis (3)	C00078,C00079,C00082

## Discussion

AMD is a complex disease. Some of the genetic factors associated with the risk of developing AMD have been identified [20], but its pathogenesis must be fully determined. Genome-wide association studies showed that the mechanism underlying AMD pathogenesis involves complement activation, phospholipid synthesis, oxidative stress, and apoptosis [21]. The effects of genetic mutations and environmental changes on the body were reflected by metabolic changes. Many studies also confirmed that lipids, including phospholipids, sphingolipids, cholesterol, lipid protein, and docosahexaenoic acid, play a role on the development and progression of AMD, and the levels of some of these lipids might be higher in patients with AMD compared with those in healthy individuals [22, 23]. Lipid metabolism is also one of the most promising biomarker candidates for AMD [24]. Differences in the metabolite levels between AMD patients and controls are detectable even during early stages of the condition [14]. For this reason, we conducted a nontargeted metabolomic profiling to study patients with wet AMD in China using a comprehensive analysis of metabolic profiles.

Research has indicated that the physiological and pathological changes of many plants and microorganisms are usually accompanied by abnormal changes in metabolic processes [25, 26]. However, these usually only involve changes in the level of expression of metabolites. In this way, screening landmark metabolites from the vast amounts of metabolomics data and establishing accurate discriminant models are significant for the early diagnosis and determination of prognosis of diseases and for the type of physiological process and period discrimination. The current work indicated that different metabolites and metabolic pathways are likely to play an important role in AMD pathophysiology. PCA, PLA-DA, and hierarchical clustering analysis indicated significant differences between endogenous metabolites of the two groups. From the data of a diverse set of metabolites detected using the UHPLC-Q-TOF MS technique, we concluded that the plasma metabolites differed between wet AMD patients and controls. Although not all different metabolites were identified, most of the features detected in this study precluded systematic structural identification. We also searched the database to help identify possible metabolites and metabolic pathways associated with wet AMD. Under the cationic mode, a total of 5443 ion peaks were tested. When the VIP score was set >1 as the selection criterion, 864 metabolites were preliminarily screened between groups. The top 10 different metabolites, particularly amino acids, were identified. For example, tyrosine, which is part of a peptide associated with melanin, neurotransmitters, and hormones, may undergo phosphorylation, sulfation, or nitration and so influence protein function. The mutations in complement factor H, which is a tyrosine-sulfated protein, have been confirmed to affect AMD development [27, 28]. Arginine had beneficial effects on endothelial dysfunction for alleviating chronic inflammation of AMD [29]. In cases of bevacizumab- and ranibizumab-resistant wet AMD, hydrocortisone is needed to treat macular intraretinal fluid in the eyes [30]. Biliverdin was produced by heme oxygenase-1, which has been found to be associated with the pathogenesis of AMD [31]. Some of our research results in metabolite changes in wet AMD patients, including amino acids level, were consistent with the results of previous studies [13, 14].

We used metabolites whose expression levels differed between the two groups to search matching KEGG human metabolic pathways that are likely to have a biological association with AMD. A total of 7 metabolic pathways included ≥5 different metabolites; in particular, biosynthesis of amino acid pathways included seven different metabolites. New global biomarkers and gene expression signatures of AMD were found, further indicating that inflammation was a feature of AMD etiology and that it involves genetics, environment, and stochastic factors [32]. Our results further identified the main metabolic pathways related to AMD. These results provide useful information for elucidating the pathogenesis of AMD and the discovery of AMD biomarkers.

Different metabolites can indicate the key points of overall metabolism in the body’s metabolic pathway and changes in substance and function after abnormal metabolic networks appear because of disease pathology. In this study, most of the different metabolites were amino acids, such as tyrosine, phenylalanine, methionine, and arginine, indicating that AMD is a disorder of amino acid metabolism. Changes in these amino acids can distinguish wet AMD patients from controls. We plan to examine the targeted metabolomic platform further through validation experiments and exact qualitative and quantitative analyses of screening metabolites [33]. One goal of these metabolomic studies is to search for AMD-specific biomarkers that may serve as effective tools that can be used to determine a given patient’s risk of developing AMD, and these biomarkers represent promising new targets for AMD-directed diagnostics and therapeutics.

## Conclusions

In conclusion, these data revealed that certain metabolites differed between wet AMD and controls. Pathway analysis also suggested that certain metabolic pathways involved into the the pathophysiology of AMD. These findings may provide promising new targets for AMD-directed therapeutics and diagnostics.

## Abbreviations

AMD: Age-related macular degeneration
ESI: Electrospray ionization
FC: Fold Change
KEGG: Kyoto Encyclopedia of Genes and Genomes
PCA: Principal component analysis
PLS-DA: Partial least squares-discriminate analysis
QC: Quality control
UHPLC-Q-TOF MS: Ultra-high-pressure liquid chromatography and quadrupole-time-of-flight mass spectrometry
VIP: Variable importance for the projection
